# Effect of acute inflammatory reaction induced by biopsy on tumor microenvironment

**DOI:** 10.1007/s00432-024-05704-7

**Published:** 2024-04-05

**Authors:** Yuanyuan Chen, Hualian Liu, Yadong Sun

**Affiliations:** 1 Department of Stomatology, The Third Affiliated Hospital of Soochow University, Changzhou, China; 2 Department of General Practice, Unit 94587 of the Chinese People’s Liberation Army, Lianyungang, China

**Keywords:** Biopsy, Inflammation, Immune cells, Tumor immunosuppressive microenvironment, Tumor immunotherapy

## Abstract

When it comes to the diagnosis of solid tumors, biopsy is always the gold standard. However, traumatic and inflammatory stimuli are so closely related to tumor initiation and development that the acute inflammatory response induced by biopsy can give rise to changes in the tumor microenvironment, including recruitment of immunosuppressive cells (M2 macrophages, Treg cells, Tumor-associated neutrophils) and secretion of inflammation-associated cytokines, to create immunosuppressive conditions that enable the increase of circulating tumor cells in the peripheral circulation and promote the metastatic spread of tumors after surgery. In this review, we discuss dynamic changes and inhibitory characteristics of biopsy on tumor microenvironment. By investigating its mechanism of action and summarizing the current therapeutic strategies for biopsy-induced tumor immunosuppressive microenvironment, the future of using biopsy-induced inflammation to improve the therapeutic effects and prognosis of patients is prospected.

## Introduction

Cancer is the second leading cause of human death (Kocarnik et al. 2022), with 23.6 million new cancer cases and 10 million cancer deaths in the latest cancer incidence statistics for 2019. As we all know, the early diagnosis of cancer is critical. Histopathology is currently the gold standard to identify any suspicious lesion, where biopsy is essential for correct diagnosis, prognosis, and determination of individualized treatment based on tumor gene profile and biomarkers (Ho et al. 2020). When it comes to uncertain suspicious lesions, biopsy has always been considered the optimal choice for minimal invasion (less trauma) and definitive pathological diagnosis. Current methods of biopsy include cutting biopsy, resection biopsy, needle biopsy, and so on (Hobson et al. 2013). However, there is growing evidence that even minor surgical trauma can affect several pathological and physiological processes, which may promote postoperative metastatic spread and tumor recurrence. Local effects caused by biopsy include tumor seeding and wound healing responses that enable the migration, proliferation, and differentiation of tumor cells, extracellular matrix remodeling, angiogenesis, and extravasation (Alieva et al. 2017, 2018; Al-Sahaf et al. 2010; Peeters et al. 2006; Lee et al. 2009).

Between chronic inflammation, wound healing, and cancer, There are numerous connections (Karin and Greten 2005). For example, ulcers and chronic inflammation can exacerbate the progression of pre-neoplastic cells to cancer (Antonio et al. 2015; Heuckmann and Thomas 2015; Coussens and Werb 2002). In normal acute inflammatory conditions, the inflammatory response is self-limiting after tissue damage or infection, with immune cells breaking down by apoptosis or returning to circulation (Shaw and Martin 2009). Malignant tissues enhance pro-inflammatory signals to reach tumor demands (Chang et al. 2004), resulting in excessive healing (Schäfer and Werner 2008). Relevant studies (Al-Sahaf et al. 2010, Coffey et al. 2003, Demicheli et al. 2008, Ceelen et al. 2014, Thaker et al. 2006, Troester et al. 2009) have shown that surgical incisions, including biopsies, are associated with higher rates of local recurrence and elevated rates of lymph node metastasis. At the same time, the wound caused by biopsy can trigger an acute inflammatory reaction, which can promote the change of the tumor microenvironment, just like chronic inflammation can promote the growth and metastasis of the tumor.

Tumor microenvironment (TME) refers to the presence of non-cancer cells and their components, including the molecules they produce and release, in tumors. Among them, there are a series of complex interactions between immune cell types and tumor cells, which affect tumor progression, invasion, and metastasis (Hinshaw and Shevde 2019). Biopsy induced an immunosuppressive TME. The tumor immunosuppressive microenvironment promotes malignant progression by promoting tumor immune evasion, angiogenesis, and metastasis (Quail and Joyce 2013; Mathenge et al. 2014). This review aims to summarize the impact of biopsy-induced acute inflammation on the tumor microenvironment, to investigate whether the associated anti-inflammation and immunotherapy can inhibit biopsy-induced tumor cell migration and proliferation, and to explore potential strategies for circumventing biopsy-induced adverse reactions.

## Tumor microenvironment

### Tumor-associated neutrophil (TAN)

Besides tumor cells, stromal cells, blood vessels, and infiltrating inflammatory cells are the main components of the tumor microenvironment (Mantovani et al. 2008). There is a profound interaction between tumor cells and neutrophils to prove that TAN is a crucial player in the tumor microenvironment (Galdiero et al. 2013). Tumors produce factors such as granulocyte colony-stimulating factor (G-CSf) and granulocyte–macrophage colony-stimulating factor (GM-CSF), which promote the release of neutrophil from the bone marrow and secrete growth factors to extend their life span (Sionov et al. 2015).

**Fig. 1:**
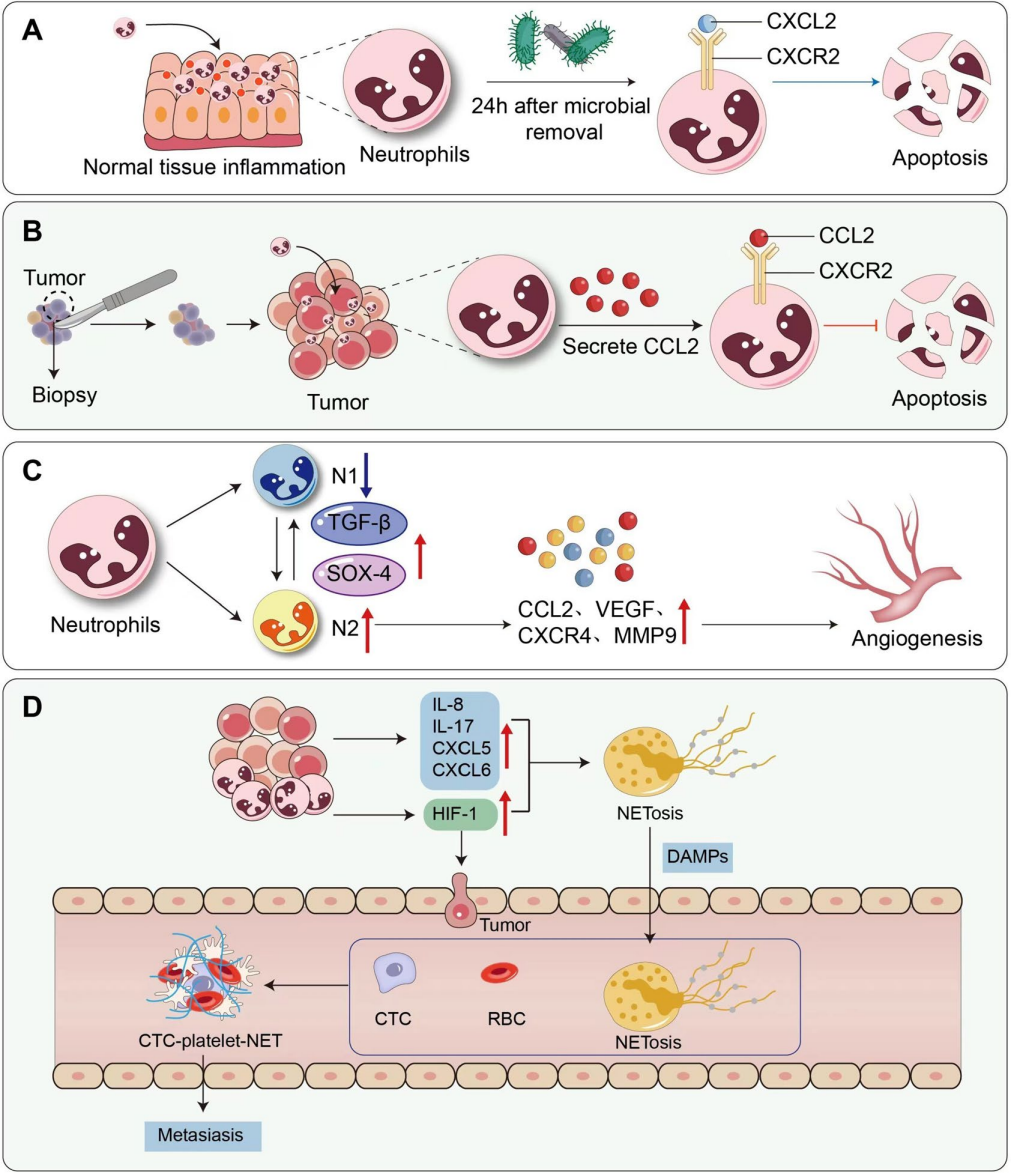
Mechanism of tumor promotion in acute inflammation induced by biopsy. **A** In normal tissues, under external stimulation, the body recruits neutrophils through DAMP, and at 24 h after microbial removal, neutrophils produce CXCL2 signals through CXCR2 to induce neutrophil apoptosis. **B** In the post-biopsy tumor tissue, CCL2 mediated through neutrophil receptor CXCR2 prevents neutrophil apoptosis. MMP-9 secreted by neutrophils processed CXCL5 further promotes neutrophil recruitment. **C** After the biopsy, TAN polarized N1 to N2, and the N2 phenotype promotes angiogenesis and tumor spread by expressing ARG, CCL2, CCL5, VEGF, CXCR4, and MMP-9. **D** Sustained inflammatory induction causes the release of Pro-inflammatory cytokines IL-8, Il-17, G-CSF, CXCL5, and CXCL6 from tumor cells, stimulating TAN to develop NETosis. Moreover, HIF-1 stimulates proteolytic enzymes in the NET to loosen the ECM and capillary walls, promoting the infiltration of cancer cells. NET can also wrap CTCs with platelets and form NET-platelet-CTC aggregations to help distant metastasis of tumors

During an inflammatory response, the body increases the number of neutrophils in the peripheral blood by amplifying granule production in the bone marrow, which signals the recruitment of neutrophils, known as Damage-associated molecular model damps (DAMP). With the help of the G protein-coupled receptor directly activating neutrophils (Wheeler et al. 2009), the release of neutrophils and activation of immune cells in resident tissues can kill invading microbes. Neutrophils, as the first inflammatory cells recruited, migrate to the wound in response to chemoattractants released from platelets as well as chemokines present on the surface of endothelial cells. 24 h after the removal of microorganisms from most injuries, some neutrophils would face apoptosis (Yager and Nwomeh 1999), but biopsy, as an acute inflammatory caused by surgical trauma, enriches neutrophils. Through the mediation of neutrophil receptor (CXC-chemokine receptor 2, CXCR2), Chemokine ligand 2 (CCL2) prevents neutrophil apoptosis (Yang et al. 2012) (Fig. 1A, B). Neutrophil Matrix metalloproteinase 9 (MMP-9) can handle the CXC motif chemokine 5 (CXCL5) to promote further the neutrophil recruitment (Song et al. 2013), angiogenesis, and tumor cells’ migration in vivo (Qian et al. 2011; Shang, et al. 2012). In a recent glioblastoma multiforme study, neutrophils, as a vehicle, were indirectly involved in biopsy-induced tumor cell migration by recruiting mononuclear macrophages into the tumor. This study has been tested by systemic neutrophil depletion injection using Ly6G antibodies to prevent biopsy-induced tumor cell migration (Chen et al. 2022). Similarly, in vivo, biopsy induced a neutrophil-dependent increase in the motility of glioma tumor cells in mice. In addition, CCL2, a chemokine secreted by tumor cells and stromal cells, mediates the recruitment of monocytes and neutrophils, which verified that blocking CCL2 can reduce the percentage of glioblastoma migration (Chen et al. 2022).

Similar to Tumor-associated macrophage (TAM), neutrophils may be induced to a tumor-promoting (N2 neutrophils) or anti-tumor (N1 neutrophils) phenotype. N1 neutrophils produce more superoxide and hydrogen peroxide and express higher levels of FAS, TNF-α, CCL3, and intercellular cell adhesion molecule-1 (ICAM-1). However, levels of Arginase (ARG), CCL2, CCL5, vascular endothelial growth factor (VEGF), CXCR4, and MMP-9 were lower than those of N2 neutrophil, characterized by upregulation of chemokines CCL2,3,4,8,12,17 and CXCL1,2,8,16 (Fridlender and Albelda 2012). Transforming growth factor-β (TGF-β) in the tumor microenvironment induces TAN populations with a pro-tumor phenotype (Fridlender et al. 2009), while interferon-β(IFN-β) can promote the N1 phenotype (Jablonska et al. 2010). Edward Gitau Mathenge et al. showed that in breast cancer incision biopsy, the expression of TGF-β and SRY-Box Transcription Factor 4 (SOX4) was significantly increased in tumors after biopsy, participating in the SOX4/EZH2 EMT pathway initiated by TGF-β (Mathenge et al. 2014). At the same time, the increase of circulating tumor cells (CTC) promotes the rise of lung metastasis of breast cancer after biopsy, which forms an immune microenvironment that suppresses immunosuppression and promotes metastasis. It was confirmed that biopsy increased the expression of TGF-β in the tumor microenvironment, thereby inhibiting neutrophil activity and cytotoxicity and increasing the percentage of N2 neutrophils (Fig. 1C).

Neutrophils are the first response to surgical trauma. They can cause neutrophils extracellular traps (NET), a fishnet-like structure that can capture microbes invading blood and tissues (Demicheli et al. 2008), but the production of NET promotes cancer progression. Sustained inflammatory induction causes the release of Pro-inflammatory cytokines IL-8, Il-17, G-CSF, CXCL5, and CXCL6 from tumor cells and recruitment of neutrophils from the bone marrow to the tumor area, stimulating TAN to develop NETosis. Moreover, hypoxia-inducible factor (HIF-1), one of the neutrophil transcription factors (McInturff et al. 2012), stimulates proteolytic enzymes in the NET to loosen the extracellular matrix (ECM) and capillary walls, promoting the infiltration of cancer cells. NETosis is a defense system, but excessive NET produced after overstimulation caused by biopsy, which is rich in many proteolytic enzymes, can lead to local invasion of cancer cells through degradation of ECM (Dam et al. 2019). At the same time, NET can also wrap CTCs with platelets to evade immune cell attacks and form NET-platelet-CTC aggregations (Najmeh et al. 2017) to help distant metastasis of tumors (Fig. 1D). Meanwhile, neutrophils can also promote tumor cell survival through NK cell inhibition, and stimulate tumor cell extravasation through IL-1 and MMPs secretion (Spiegel et al. 2016).

### Tumor-associated macrophage (TAM)

Macrophages are monocytes with phagocytic properties found in tissues and can be divided into M1 and M2 types according to their degree of differentiation and function (Mantovani and Locati 2013). M1 macrophages are pro-inflammatory and promote anti-tumor immune responses by producing inflammatory cytokines such as IL-12, IL-23, IFN-γ, and reactive oxygen species. By secreting immunosuppressive cytokines such as IL-10 and TGF-β, M2 macrophages have the characteristics of promoting tumor, including remodeling of the extracellular matrix, promotion of tumor cell invasion, and metastasis, angiogenesis, lymphangiogenesis, formation of a TME that favors immunosuppression, which is conducive to tumor progression (Mantovani et al. 2013, 2002).

The typical interferon regulatory factor/signal transducer and activator of transcription (IRF/STAT) signaling pathway is activated by IFN and toll-like receptor (TLR) signaling pathways, tilting macrophage function toward the M1 phenotype by signal transducer and activator of transcription 1 (STAT1) or toward the M2 phenotype by IL-4 and IL-13. The balance between activation of STAT1 and STAT3/STAT6 regulates macrophage polarization and activity finely. Biopsy breaks this balance. The predominance of NF-κB (Hagemann et al. 2008) and STAT1 activation promotes polarization of M1 macrophages, leading to cytotoxicity and inflammatory function. In contrast, after the biopsy, the predominance of STAT3 (Liu et al. 2008) and STAT6 activation leads to polarization of M2 macrophages, which is associated with immunosuppression and tumor progression (Sica and Bronte 2007). Furthermore, the progression and metastasis of tumors are associated with the transformation of regional lymph nodes toward M2 polarization (Wehrhan et al. 2014). This also means that the biopsy promotes the result of regional lymph node metastasis. More and more studies have confirmed that the ratio of M2 to M1 of tumor-associated macrophages is associated with poor prognosis in tumors, including ovarian cancer, gastric cancer, and squamous cell carcinoma of the head and neck (Yuan et al. 2017; Yang et al. 2019; Troiano et al. 2019).

Recruitment and invasion of inflammatory cells and repolarization of resident tissue macrophages to type M2 are primary immune responses after the biopsy (Sica and Mantovani 2012). In an immunohistochemical study, there was an increase in M2 polarization in samples obtained during oral squamous cell carcinoma (OSCC) tumor resection. Biopsy-induced tissue trauma may explain the observed metastasis of macrophage polarization toward tumor-promoting type M2 and may lead to an acceleration of tumor progression. Biopsy triggers an acute inflammatory response and initiates the wound healing process (Hobson et al. 2013), initially a critical inflammatory reaction dominated by M1 macrophages, followed by the transformation from macrophage polarization to M2 macrophages (Mantovani et al. 2013). M2 macrophages promote tumor progression by VEGF and extracellular matrix remodeling proteins (such as MMP). And they secrete the immunosuppressive cytokines IL-4, IL-10, and TGF-β to induce T cell tolerance. In another study, a significant increase in TAM was observed in recurrent tumors, mainly with an M2 phenotype, exhibiting immunosuppression that promoted tumor progression, which means TAM is associated with recurrence after tumor resection. Since M2 macrophages express the chemokines CCL17, CCL22, and CCL24 (Mantovani 2008; Martinez et al. 2006), and chemokines can also affect the polarization of macrophages, CCL2 and CXCL4 drive macrophages to form an M2-like phenotype (Gleissner et al. 2010; Roca et al. 2009). After the biopsy, tumor tissue induces migration and proliferation of tumor cells by the recruitment of CCL-2-dependent macrophages (Qian et al. 2011; Chen et al. 2022) and increases migrating motor tumor cells. The movement throughout the day did not direct to the biopsy site, which means that the observed trend isn’t due to wound healing but to the enhanced motility of tumor cells (Alieva et al. 2017; Dal-Secco et al. 2015).

**Fig. 2:**
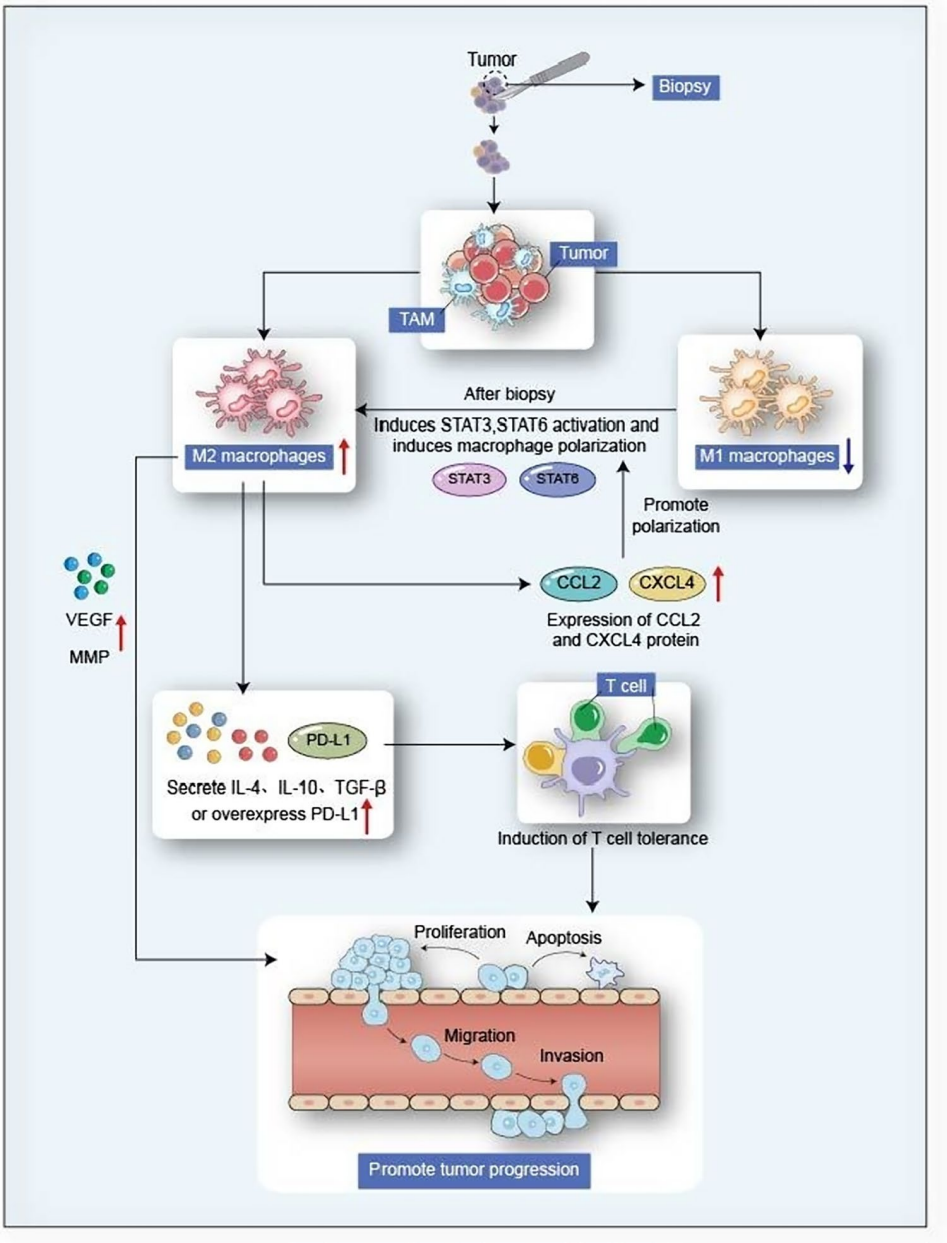
After the biopsy, M2 macrophages are polarized toward M1 by activating STAT3 and STAT6. Moreover, chemokines such as CCL2 and CXCL4 secreted by the M2 type promote M2 polarization. On the one hand, M2 promotes angiogenesis through the secretion of VEGF and MMP, and on the other hand, it induces T cell tolerance through IL-4, IL-10, TGF-β, and the increase of PD-L1 expression on macrophages

Under systemic inflammation induced by surgical stress, the expression of programmed cell death ligand 1 (PD-L1) on activated macrophages exceeds that of tumor cells, indicating that TAM-associated PD-L1 plays a prominent role in immunosuppression and tumor growth (Sun et al. 2017). At the same time, the pro-inflammatory chemokines secreted by TAM have been shown to drive the behavior of malignant tumor cells, and the newly recruited TAM after biopsy may arise from the blood-resident monocytes infiltrating the tissue. However, more in vivo studies are needed to investigate further the exact nature of biopsy-induced macrophages and their recruitment mechanisms. (Fig. 2).

### T regulatory cells (Treg cells)

As a subgroup of inhibitory T cells, Treg cells not only inhibit abnormal immune responses against self-antigens but also inhibit anti-tumor immune responses. Treg cells can regulate T cells and control B cells, natural killer (NK) cells, dendritic cells (DC), and macrophages through humoral and intercellular contact mechanisms. Treg cells can also participate in Treg-mediated tumor immunosuppression through a variety of molecules, including cytotoxic T-lymphocyte-associated protein 4 (CTLA-4), IL-2, IL-10, TGF-β, IL-35, glucocorticoid-induced tumor necrosis factor receptor (glucocorticoid TNF receptor), lymphocyte activation gene 3 (LAG3), granzyme B, adenosine, and cyclic adenosine monophosphate (CAMP6) (Tanaka and Sakaguchi 2017; Sakaguchi et al. 2008). In experimental models of lung cancer, tumors that had undergone partial tumor resection were found to be infiltrated with alternatively activated macrophages and Tregs by immunosuppression, which prevented the recruitment of CD8^+^T lymphocytes into the tumor and contributed to faster tumor recurrence. High expression of TGF-β and cyclooxygenase 2 (COX2) at the resection site stimulates the infiltration of these immunosuppressive cells. COX2 expression in tumors facilitates the transformation of nascent naive CD4^+^T cells into Foxp^3+^Tregs, as well as their recruitment through the interaction between COX2-stimulated prostaglandin E2 (PGE2)(a signal for PGE2 to drive wound inflammation-mediated proliferation of pre-neoplastic cells (Antonio et al. 2015)) and Treg-expressed recombinant prostaglandin E receptor 2 (EP2) (Karavitis et al. 2012). This behavior promotes immunosuppression. Moreover, the increase of N2-type neutrophils induced by biopsy encourages neutrophils to recruit regulatory T cells into the tumor by producing CCL17, creating an immunosuppressive environment (Mishalian et al. 2014).

### Other immunosuppressive cells and related factors

Changes in the tumor microenvironment after biopsy also include the upregulation of many vital inflammatory cytokines and mediators, including recombinant S100 calcium-binding protein A8 (S100A8), CXCL1, CXCL2, IL-1β, TNF-α, and COX2, which activate an inflammatory signaling cascade (including MyD88 and the transcription factor NF-κB), that are responsible for recruitment of multiple inflammatory and immune cell populations, including neutrophil, macrophages, myeloid suppressor cells (MDSC), and Tregs (Table 1).

**Table 1:** Other immunosuppressive cytokine and possible inhibition mechanism

Cytokine	Inhibition mechanism	References
SOX4	SOX4 directly regulates the expression of enhancer of zest homolog 2 (EZH2), which encodes Polycomb Histone methyltransferase and epigenetically modifies the expression of several epithelial-to-mesenchymal transition (EMT) genes, promoting gene expression changes of EMT-related in the remaining tumor cells and immune escape. Biopsy enhanced the expression of genes involved in the development of EMT and the expression of the downstream EMT genes SNAI 2 (Slug), ZEB 2, and CDH2 (N-cadherin) Ezh 2. SOX4 is associated with a metastasis-related poor outcome in early node-negative patients. Moreover, the EMT-related gene expression changes were also significantly associated with elevated CTC. This transition from epithelial to mesenchymal state that tumor cells can undergo during biopsy is necessary for tumor cell migratory behavior	(Alieva et al. 2017; Mathenge et al. 2014; Zhang et al. 2012; Yu et al. 2013)
TGF-β	The increase of TGF-βstimulates the upregulation of SOX-4, the primary EMT regulator, and initiates the SOX4/EZH2 EMT pathway. TGF-β induces TAN population with tumor-promoting phenotype	(Fridlender et al. 2009; Laoui et al. 2011; Predina et al. 2013)
COX2	COX2 stimulates the production of PGE2 and recruits Foxp3 + Tregs expressing EP2 into tumors via the PGE2/EP2 interaction-dependent pathway. Expression promotes the transformation of nascent naive CD4^+^T cells into FoxP^3+^ Tregs	(Karavitis et al. 2012; Predina et al. 2013)
TNF-α	TNF-α produced by tumor cells or inflammatory cells in the TME can promote tumor cell survival by inducing NF-κB-dependent anti-apoptotic molecules. TNF-α has also been shown to promote angiogenesis and induce the expression of VEGF and HIF-1αin tumor cells	(Luo et al. 2004; Sainson et al. 2008; Jing et al. 2011
IL-6	IL-6 is a pro-inflammatory marker and may not be a carcinogenic agent by itself. Still, in the context of inflammation induced by biopsy, it may be a critical factor in promoting the formation of cancer cells’ seeding and metastatsis in peripheral tissues. Although IL-6R expression is mainly restricted to leukocytes and hepatocytes, the presence of IL-6-associated soluble IL-6R (sIL-6R) can trigger IL-6 trans-signaling pathways in almost any cell, including tumor cells. In mice with defects in IL-6, the number of tumors caused by acute inflammation is also reduced	(Rincon 2012; Dethlefsen et al. 2013; Tzeng et al. 2013)
S100A8	Increased expression of S100A8 is associated with chemotactic recruitment of MDSC and increased vascular permeability. It also activates p38 MAPK, ERK1/2, and transcription factor NF-kB	(Yu et al. 2014; Vogl et al. 2018)

The MDSCs recruited after the biopsy are a heterogeneous population of bone marrow cells that inhibit anti-tumor immune responses and can suppress T cells, NK cells, and DCs, stimulating immune regulatory factors, as with Tregs and TAMs. The pro-angiogenic role of MDSC is partly driven by BV8, upregulateing G-CSF through a STAT3-dependent mechanism (Shojaei and Ferrara 2008; Shojaei et al. 2007). At the same time G-CSF may further promote tumor angiogenesis by inducing neutrophils to produce VEGF-A (Ohki et al. 2005). Traumatic surgery (such as biopsy) induces MDSC amplification produce angiogenic factors and matrix-degrading enzymes, such as VEGF and MMP-9, to promote tumor angiogenesis (Murdoch et al. 2008), allowing it to form a microenvironment that promotes tumor metastasis after biopsy.

Biopsy stimulates the release of VEGF, platelet-derived growth factor (PDGF), prostaglandins, TGF-β, coagulation factors, and complement, enhancing new angiogenesis required for tumor growth (Thaker et al. 2006; Hormbrey et al. 2003). Although these mediators are of great value for wound healing, they contribute to the rapid expansion of Tregs, MDSC, and angiogenic factors in the context of recurrent tumors. Among the many mediators used in biopsy wound healing, transforming growth factors and alkaline fibroblast growth factors have been shown to increase neoangiogenesis due to tumor growth and healing mechanisms significantly. These growth factors can be secreted directly by tumor cells because their transcriptomes are changed, inducing tumor cell proliferation.

Human breast cancer biopsies have also been found to induce eosinophil granulocyte recruitment and enhance the proliferation of adjacent cancer cells. Eosinophil granulocyte is part of the inflammatory response during wound healing and promotes tissue regeneration by secreting various granular cytokines (Spencer et al. 2014). The proliferation frequency of tumor cells near the biopsy wound is increasable, but the potential correlation with prognosis is uncertain, and the mechanism remains unclear.

## Potential therapeutic strategies

The acute inflammatory reaction induced by biopsy is a congenital reaction that promotes dynamic changes in the tumor microenvironment. Solid tumors will grow and progress during the particular period after biopsy. As a result, appropriate treatment measures should be taken, which should take the balance of surgery-induced inflammation, wound healing, and tumor metastasis into account. Many chemokines, growth factors, cytokines, and immune cells are involved in the acute inflammatory response after biopsy and during wound healing. Therapeutic strategies that target anti-inflammatory signals released after surgical trauma, reverse macrophage polarization, and immunosuppressive cells (such as MDSC or Tregs) may have higher efficacy.

### Anti-inflammatory therapy

Since the inflammatory stimulation induced by biopsy can induce the proliferation, invasion, and metastasis of tumor cells, it is essential to prevent the inflammatory stimulus and eliminate the adverse reaction. Some studies have demonstrated that anti-inflammatory therapy can significantly reduce the risk of metastasis associated with wound healing in primary tumors. For example, using non-steroidal anti-inflammatory drugs such as ibuprofen only in the first three days after biopsy further inhibits the synthesis and release of PGE-2 by inhibiting COX2 and reduces the recruitment of Treg cells in tumors. It also helps to reduce the metastatic number of tumor cells. Maria Alieva et al. found that biopsy-like lesions in GBM induced the migration and proliferation of tumor cells through CCL2-dependent recruitment of macrophages. Through blocking the recruitment of macrophages, which means the local injection of anti-inflammatory antibodies at the biopsy site or administration of dexamethasone three days before biopsy, the inflammatory responses were suppressed and biopsy-induced tumor Progression was blocked (Alieva et al. 2017; Imaizumi et al. 2009; Goswami et al. 2017), which reduced the mice’ levels of circulating monocytes and other immune cells. Also, these were validated in human specimens and the specificity of a retrospective analysis of biopsies in 10 patients required a larger biopsy volume and a higher degree of malignancy (the cells themselves have an intrinsic ability to migrate), pre-treatment with dexamethasone for prevention (Wong et al. 2015; Pitter et al. 2016). A way to harness biopsy-induced inflammation to treat cancer is to combine biopsy with therapies that can mitigate immunosuppression in the tumor microenvironment while enhancing both innate and adaptive tumor-reactive immune responses (Winter et al. 2017). However, the mechanism of dexamethasone's effect on macrophage polarization is not yet known. Since anti-inflammatory drugs also directly inhibit tumor growth (Zelenay et al. 2015), it was found that meloxicam treatment alters the TAM phenotype in mice before injection of breast cancer cells. Before meloxicam administration, surgical injury induces upregulation of CD206 on the surface of TAM, suggesting that M2 polarization is generally associated with immunosuppressive properties. The treatment of meloxicam in mice can prevent the increased expression of CD206 and result in decreased expression of PD-L1 on tumor-associated macrophages. At the same time, local injection of anti-inflammatory antibodies at the biopsy site immediately after surgery is also an alternative strategy to inhibit biopsy-induced tumor cell migration and proliferation (Predina et al. 2013).

### Immunotherapy and combination therapy

According to the dynamic changes and inhibition of the tumor microenvironment, immunotherapy targeting the Pro-inflammatory cytokine induced by acute inflammation and immunosuppressive cells is also one of the current therapeutic strategies. Some anti-cytokine (Balkwill and Mantovani 2012) has been used in the treatment of cancer. For example, the blocking of CCL2 can inhibit metastasis of tumors (Alieva et al. 2017) and interrupt the CXCR4/CXCL12 chemokine axis, which can be used to sensitize drug-resistant tumor cells to chemotherapy or radiotherapy and may inhibit angiogenesis and proliferation of tumor cell. In a phase II trial of chimeric antibodies against IL-6 in ovarian cancer, plasma levels of chemokines that promote immune cell recruitment (CCL2 and CXCL12) and angiogenesis (VEGF) were significantly reduced (Anglesio et al. 2011). Thea L Rogers et al. found that bisphosphonates could inhibit macrophage proliferation, migration, invasion, and induce apoptosis, as well as serve as one of the therapeutic methods to inhibit the proliferation of type M2 macrophages after biopsy (Rogers and Holen 2011). Using a mouse model of breast cancer, Coscia et al. investigated the cellular effects of clinically achievable zoledronic acid (ZOL) dose-induced reduction in VEGF levels, which is one of the most important factors to induce the phenotypic polarization of macrophages from M1 to M2. Therefore, the use of ZOL before biopsy helps to restore the M1 phenotype with a view of achieving the effect of hindering the progression of tumor(Coscia et al. 2010). Similarly, in a similar study, celecoxib, a selective COX2 inhibitor, was found to change the TAM phenotype from M2 to M1, and the expression of the M1-associated cytokine IFN-γ was significantly upregulated (Na et Al. 2013). They orient it to the M1 phenotype even in the presence of M2-polarized cytokines such as IL-4, IL-13, and IL-10. Furthermore, MDSC inhibitors derived from targeting surgical stress, such as bone marrow stromal cell (BMSC), 5-fluorouracil, gemcitabine, and docetaxel, can all contribute to a dramatic reduction in the number of MDSC and become the key to prevent metastasis after surgery(Wang et al. 2016). The use of MDSC inhibitors reduces the risk of postoperative metastasis.

Pre-operative IL-2 therapy has been shown to counteract surgery-induced immunosuppression and prolong survival in patients with colorectal cancer (Brivio et al. 1996). Other modulators such as granulocyte–macrophage Colony-stimulating factor, IFN-α, and TNF-α also improved postoperative immune function. Although IL-2 and IFN-α treatments have shown severe side effects (Brivio et al. 2002), several recent studies have reported that short-term dosing schedules and drug delivery strategies cause only minor side effects, suggesting that progress should be made (Kutza et al. 1997).

The up-regulation of PD-L1 expression in TAM after surgical stress suggests that the blocking of PD-1 with monoclonal antibody may be an effective therapy to fix immunosuppression. In the mouse model of surgical stress, blockade of PD-1 with specific antibodies can restore the number and secretion capacity of CD8^+^T cells (Sun et al. 2017). In addition, the expression of PGE2 was significantly up-regulated after surgery, and the combination therapy of anti-PD-1 and PGE-2 inhibitors restored the dysfunction of cytotoxic T lymphocytes induced by surgery.

In addition to using TAM-related blockers, when compared with high doses, low-dose radiotherapy polarizes macrophages toward the anti-tumor M1 type (Klug et al. 2013). Local radiation can also stimulate immune responses by releasing tumor-associated antigens, promoting antigen presentation, upregulating MHC-1 complexes in tumor cells, or reducing the number of Treg cells (Davern and Lysaght 2020). Whether the combination of radiotherapy and immunotherapy can achieve higher benefits needs to be further explored.

## Conclusions

Although various studies have shown that biopsies create an immunosuppressive tumor microenvironment, by now, biopsies and histopathology remain the gold standard for a definitive diagnosis of tumors. So, the benefits of biopsy outweigh any potential adverse side effects it brings. The significant differences between individuals often mask changes in the tumor microenvironment during processes such as the recruitment of immune cells and the induction of tumor cell migration and proliferation. Moreover, the variability of the tumor microenvironment between different biopsy methods has yet to be studied, which may be due to personalized treatment. The choice of biopsy methods often depends on the site, nature, and size of the tumor. Currently, exploring the differences in the tumor microenvironment of different biopsy methods is more helpful for determining of personalized treatment. How to reduce the side effects of biopsy needs to be further explored. Given the dynamic changes and suppressive features of the tumor microenvironment in the acute inflammatory response elicited by biopsy, anti-inflammatory drugs or immunotherapies targeting molecules or signaling pathways in the TME remain to be investigated. These results open the possibility of developing alternative therapies based on local inhibition after biopsy for accurate diagnosis of lesions and avoidance of adverse reactions. Furthermore, biopsy-induced inflammation should be considered for cancer treatment. For anti-tumor effects, biopsy and immunotherapy should be combined, while enhancing both innate and adaptive tumor-reactive immune responses and promoting the formation of an inflammatory immune microenvironment. It is necessary to explore the possibilities of anti-inflammatory therapy, and immunotherapy strategies after biopsy, to improve the clinical benefit of biopsy, to explore the possibility of combination therapy and to evaluate the effectiveness of specific treatment, which requires a deeper understanding of the dynamic changes of TME after biopsy. Of course, in current relevant studies, the change of tumor microenvironment seems to be irrelevant to the time interval between biopsy and tumor resection.

## Data Availability

Reviewed studies and their results can be located at PubMed database: https://pubmed.ncbi.nlm.nih.gov/.
